# Metabolic engineering of a synergistic pathway for *n*-butanol production in *Saccharomyces cerevisiae*

**DOI:** 10.1038/srep25675

**Published:** 2016-05-10

**Authors:** Shuobo Shi, Tong Si, Zihe Liu, Hongfang Zhang, Ee Lui Ang, Huimin Zhao

**Affiliations:** 1 Metabolic Engineering Research Laboratory, Science and Engineering Institutes, Agency for Science, Technology and Research, Singapore; 2 Department of Chemical and Biomolecular Engineering, University of Illinois at Urbana-Champaign, Urbana, IL 61801, United States.

## Abstract

*n*-Butanol has several favourable properties as an advanced fuel or a platform chemical. Bio-based production of *n*-butanol is becoming increasingly important for sustainable chemical industry. Synthesis of *n*-butanol can be achieved via more than one metabolic pathway. Here we report the metabolic engineering of *Saccharomyces cerevisiae* to produce *n*-butanol through a synergistic pathway: the endogenous threonine pathway and the introduced citramalate pathway. Firstly, we characterized and optimized the endogenous threonine pathway; then, a citramalate synthase (CimA) mediated pathway was introduced to construct the synergistic pathway; next, the synergistic pathway was optimized by additional overexpression of relevant genes identified previously; meanwhile, the *n*-butanol production was also improved by overexpression of keto-acid decarboxylases (KDC) and alcohol dehydrogenase (ADH). After combining these strategies with co-expression of *LEU1* (two copies), *LEU4*, *LEU2* (two copies), *LEU5, CimA, NFS1, ADH7* and ARO10^*^, we achieved an *n*-butanol production of 835 mg/L in the final engineered strain, which is almost 7-fold increase compared to the initial strain. Furthermore, the production showed a 3-fold of the highest titer ever reported in yeast. Therefore, the engineered yeast strain represents a promising alternative platform for *n*-butanol production.

Due to increasing demand of transportation fuels and decreasing availability of crude oils, a gradual shift from oil based fuels towards alternatives and renewable fuel resources will be required in the future. *n*-Butanol is one of the highly sought-after renewable fuels for transportation. It has comparable energy density (29.2 MJ/L) to that of gasoline (32.5 MJ/L)^1^,^2^, and can be blended in gasoline at any ratio^1^. Furthermore, *n*-butanol is more hydrophobic and less corrosive than ethanol, and therefore can be compatible with current fuel infrastructure^1^. Besides, *n*-butanol is an important industrial chemical precursor^3^,^4^, that can be used as an intermediate in the production of paints, perfumes, polymers and plastics. The global market of *n*-butanol was estimated to be over $5 billion with a predicted 4.7% growth per year^4^. *n*-Butanol can be produced commercially from fossil fuels through generally expensive and environmentally unfriendly chemical routes. The biological production of *n*-butanol is traditionally carried out through the acetone-butanol-ethanol (ABE) fermentation using *Clostridia* species. However, this process has many limitations such as lack of genetic tools, undesired byproducts, low cell density, and low alcohol tolerance of *Clostridia* species^5^. Therefore, there have been extensive efforts in both academia and industry to engineer industrially friendly organisms for the production of *n*-butanol^6^,^7^,^8^,^9^.

Various attempts have been made to transfer the *Clostridial n*-butanol pathway to more suitable industrial organisms including *Escherichia coli* and *Saccharomyces cerevisiae*. This CoA-dependent pathway yielded a production titer (30 g/L) in *E. coli* after extensive engineering^10^. However, it led to very low production in *S. cerevisiae* with a titer of 100 mg/L^11^. In addition, a reversed β-oxidation pathway was engineered to produce 14 g/L of *n*-butanol in *E. coli*^12^ and 20 mg/L of *n*-butanol in *S. cerevisiae*^13^. *n*-Butanol was also produced at 92 mg/L from l-glycine in *S. cerevisiae*^14^. Although *E. coli* has been engineered to produce higher titers of *n*-butanol than *S. cerevisiae*^6^,^8^,^9^,^10^,^12^, *E. coli* is not ideal for industrial scale production^9^ whereas *S. cerevisiae* is preferred due to its high *n*-butanol tolerance^15^, phage resistance^16^, well-established genetic tools and compatibility to current industrial infrastructure^17^. Recently, we discovered an endogenous *n*-butanol pathway in *S. cerevisiae*, and it depended on catabolism of threonine in a manner similar to fusel alcohol production by the keto-acid pathway, which produced 120 mg/L of *n*-butanol from glucose by a single gene deletion *adh1*Δ^18^. Very low level of n-propanol production was observed in the *adh1*Δ strain, enabled by separation of α-ketobutyrate from cytosolic KDCs by the mitochondrial membrane^18^. The absence of *n*-propanol is an additional advantage in contrast to the similar pathway constructed in *E. coli*^19^. As there is no compartmentalization to separate α-ketobutyrate from KDCs in *E. coli*, it is unavoidable to co-produce the byproduct, *n*-propanol, the synthesis of which shares a common intermediate (α-ketobutyrate) with the *n*-butanol pathway. There are two pathways leading to the synthesis of the common intermediate α-ketobutyrate: the threonine pathway^18^,^19^,^20^ and the citramalate pathway^20^,^21^. Both pathways have been used in *E. coli* for the production of *n*-butanol and *n*-propanol^19^,^20^,^21^, and there is no report about the adoption of the citramalate pathway in yeast. Besides *n*-butanol and *n*-propanol, α-ketobutyrate is an important building block for many chemicals^22^,^23^,^24^. In agreement with our finding, the *ADH1* gene was also deleted in another study, which led to 40 mg/L *n*-butanol^25^, and further modification to improve the flux of carbon to acetyl-CoA generated a yeast strain capable of producing up to 300 mg/L *n*-butanol^25^, which is the highest reported *n*-butanol production in yeast so far. The production of *n*-butanol in this yeast is synthesized from two parallel pathways: threonine based keto-acid pathway and *Clostridia* based ABE pathway. These studies highlighted the importance of *ADH1* deletion and incorporation of synergy into the design principle of *n*-butanol producer.

In this study, we extended our previous work to further improve *n*-butanol production in *S. cerevisiae* through a combination of strategies. First, we tried to increase the production of *n*-butanol by optimizing the threonine pathway. To achieve this purpose, we showed that a mutant *HOM3* allele encoding a feedback-insensitive aspartate kinase enabled deregulation and over-production of threonine, which facilitated the production of *n*-butanol. Furthermore, overexpression and localization of the endogenous *n*-butanol pathway to the mitochondria increased *n*-butanol production compared to overexpression of the same enzymes in the cytoplasm. Second, we introduced citramalate synthase (CimA) to shorten the endogenous pathway and constructed a synergistic pathway, which significantly improved the *n*-butanol production compared to the sole overexpression of the threonine pathway. The resulting synergistic pathway was further optimized by overexpression of an additional copy of relevant enzymes identified in this study. Next, subsequent overexpression of KDC and alcohol dehydrogenase (ADH) further increased *n*-butanol production. By combining these modifications, the final engineered yeast strain produced the highest reported *n*-butanol titer in *S. cerevisiae* (835 mg/L) with a yield of 42 mg/g glucose in anaerobic glass tubes. When cultivated in a bioreactor, the strain could produce up to 1.05 g/L *n*-butanol.

## Results

### Rational engineering of an endogenous threonine biosynthesis pathway to improve n-butanol production

**Figure 1:**
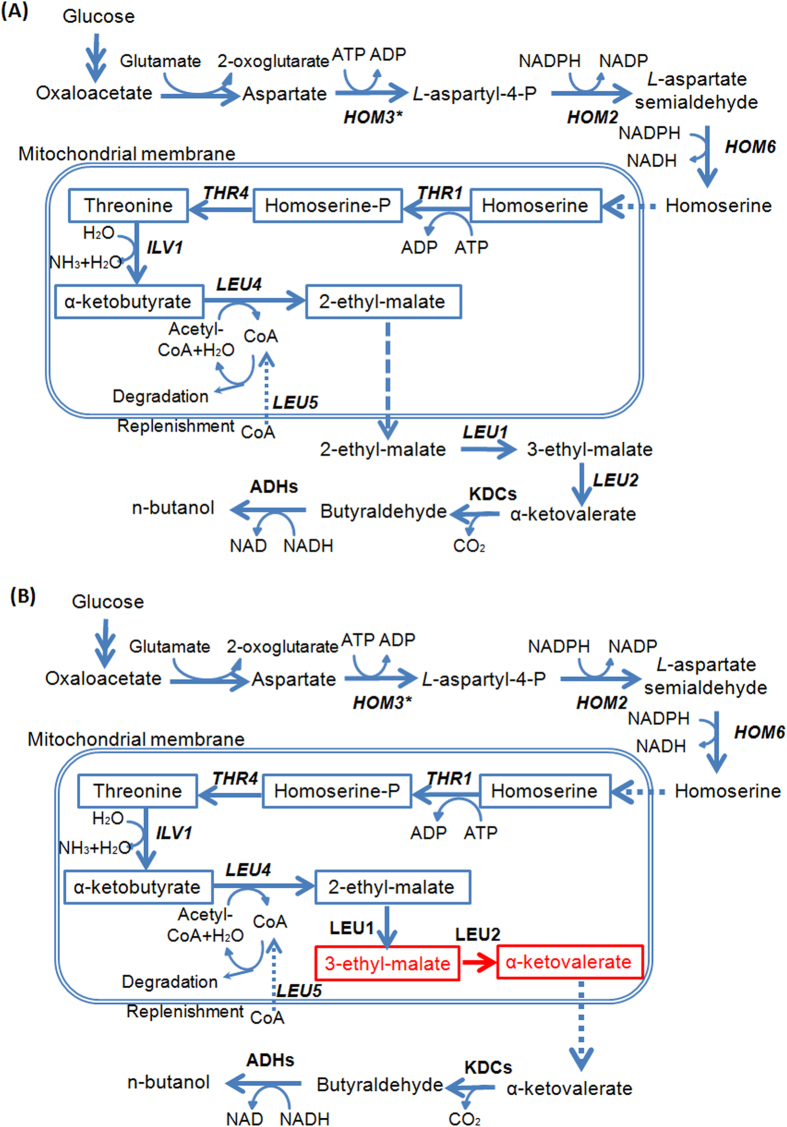
Characterization and optimization of the endogenous threonine pathway from glucose to *n*-butanol in *S. cerevisiae*. The *n*-butanol biosynthesis pathway via threonine was relocalized in cytoplasm (**A**) or mitochondria (**B**). Single and double arrows represent single and multiple enzymatic steps respectively. The broken line indicates the transport of chemicals between mitochondria and cytosol. Relocalized reactions in (**B**) were highlighted with red. The information on the biochemical pathways and enzyme locations was from previous literature^18^,^28^,^45^. L-aspartyl-4-P: 4-phospho-L-aspartate; Homoserine-P: O-phospho-L-homoserine; KDCs: keto-acid decarboxylases; ADHs: alcohol dehydrogenases.

The endogenous *n*-butanol production is dependent on the catabolism of threonine (Fig. 1A), and it was switched on by introduction of a single gene deletion *adh1*Δ with the *n*-butanol production at 120 mg/L using resting cells^18^. The maximum theoretical yield of *n*-butanol is 411 mg/g glucose via the catabolism of threonine. In this study, resting cells were cultured under micro-aerobic condition for the production of n-butanol. The initial OD of the cultures was around 1.0 after inoculation from pre-cultures, and it was maintained at a nearly constant OD during the fermentation.

**Figure 2:**
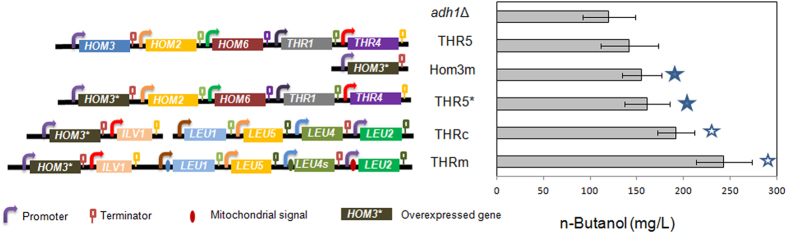
*n*-Butanol production in recombinant strains with different gene combinations. All recombinant strains were constructed in the *adh1*Δ strain. Fermentation was performed in anaerobic glass tubes in YPAD media with 20 g/L glucose under micro-anaerobic condition as previously described^18^. HOM3*: *HOM3* mutant encoding *HOM3*^G1355A^ 27; *LEU4s*: truncated *LEU4* that starts at +91 ATG^33^. Error bars indicate standard deviations of three biological replicates. A student’s t-test was used for statistical analysis of the *n*-butanol production in triplicates. Solid pentagram (★) indicates a result that was significantly different compared to the reference *adh1*Δ strain (*p* < 0.05); empty pentagram (☆) indicates a result that was significantly different compared to the Hom3m strain (*p* < 0.05).

To increase the cellular supply of threonine, five genes encoding for the enzymes responsible for converting aspartic acid to threonine (i.e., *HOM3*, *HOM2*, *HOM6*, *THR1* and *THR4*) were overexpressed on a multi-copy plasmid pRS426-THR to generate the strain THR5. There is around 19% increase in *n*-butanol production in strain THR5 (Fig. 2). However, the increase was not statistically significant. On the other hand, it has been reported that the *HOM3* gene which encodes an aspartate kinase, plays a crucial role in the regulation of the metabolic flux that leads to threonine biosynthesis^26^,^27^. In order to achieve greater threonine accumulation, strain Hom3m was constructed to carry the mutant *HOM3* allele (HOM3*) encoding a feedback-insensitive aspartate kinase in a multi-copy plasmid. As expected, the overexpression of HOM3* conferred an overproduction of *n*-butanol most likely due to an efficient supply of threonine, and it accumulated 28% more *n*-butanol than the reference *adh1*Δ strain with statistical significance. However, co-expression of HOM3* with *HOM2, HOM6, THR1*, and *THR4* (strain THR5*) did not increase the *n*-butanol production compared to strain Hom3m (Fig. 2).

Enzymes encoded by *LEU* genes (*LEU1*, *LEU4*, and *LEU2*) and *ILV1* catalyse reactions converting threonine to α-ketovalerate (Fig. 1A). Overexpression of these genes individually was demonstrated to increase *n*-butanol production^18^. Because Leu5p is involved in the accumulation of CoA in the mitochondrial matrix, and it functions together with Leu4p by providing alpha-isopropylmalate synthase activity^28^, we tested the combined expression of these beneficial genes (HOM3*, *ILV1*, *LEU1*, *LEU4*, *LEU2*, *LEU5*) in the strain *adh1*Δ. Additional overexpression of *ILV1*, *LEU1*, *LEU4*, *LEU2* and *LEU5* resulted in a strain THRc with an *n*-butanol production of 192 mg/L (Fig. 2), a further increase compared to single overexpression of HOM3*.

*n*-Butanol derived from the degradation of threonine via the keto-acid pathway takes place in the cytosol^18^,^28^, whereas the biosynthesis of threonine occurs in the mitochondria. It has been reported that compartmentalization of metabolic pathways in yeast mitochondria would affect alcohol production^29^,^30^. Therefore, we sought to relocate the threonine catabolism pathway in mitochondria to investigate its effect on *n*-butanol production (Fig. 1B). To achieve this, three N-terminal mitochondrial localization signals were designed based on previous studies, including *CoxIV* mitochondrial localization signal (CoxIVm)^30^, *CYB2* mitochondrial localization signal (CYB2m)^31^, and *CAT2* mitochondrial localization signal (CAT2m)^32^. As mitochondrial targeting sequences were not clearly defined, we tested the localization ability of these three signals using green fluorescent protein (GFP) as a reporter. These three mitochondrial signals were separately fused to the N-terminus of GFP. A strain expressing GFP without a mitochondrial signal was also constructed to distinguish cytosolic localization from mitochondrial localization. As shown in Supplementary Fig. S1, distinct localizations of the GFP in the mitochondria were observed for all three constructs, indicating that all three mitochondrial signals, CoxIVm, CYB2m, and CAT2m, were efficient in directing enzymes into the mitochondrion. Then, we targeted the downstream enzymes of threonine catabolism to the mitochondrion using these mitochondrial signals (Fig. 1B). CoxIVm and CYB2m were fused to the N-terminus of Leu1p and Leu2p respectively. It was hypothesized that *LEU4* was capable of producing two forms of α-isopropylmalate synthase by the use of alternative transcription initiation sites^33^, and only the larger of the two forms would be imported into the mitochondrion. The short form was believed to be made from transcripts starting downstream at the +91 ATG of the long form. Therefore, we fused CAT2m to the N-terminus of the short form Leu4p to direct as much of the produced α-isopropylmalate synthase into the mitochondrion as possible. As shown in Fig. 2, relocalization of Leu1p, Leu2p, and Leu4p in the mitochondria resulted in a strain THRm with a further improvement on *n*-butanol production (243 mg/L).

### Addition of the citramalate synthase mediated pathway to *S. cerevisiae* results in significantly improved *n*-butanol production

**Figure 3:**
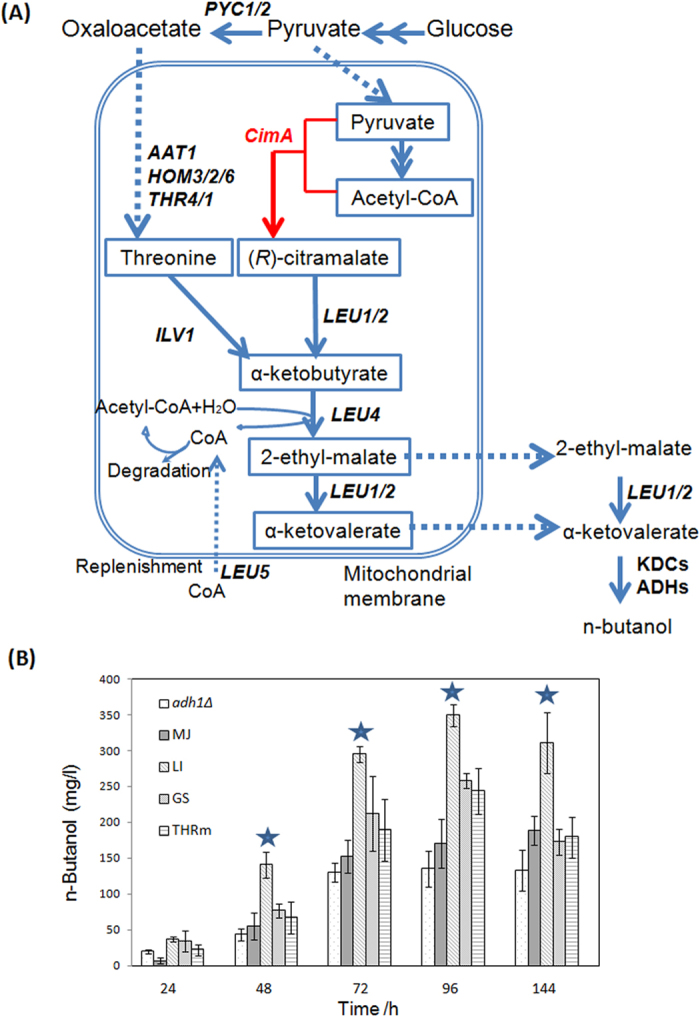
Characterization and optimization of the synergistic pathway for *n*-Butanol production by introduction of a CimA mediated pathway. (**A**) The synergistic pathway for *n*-Butanol production via the endogenous threonine pathway and a introduced CimA mediated pathway. Single and double arrows represent single and multiple enzymatic steps respectively; red arrows represent heterologous pathways; overexpressed genes are marked with red colour. KDCs: keto-acid decarboxylases; ADHs: alcohol dehydrogenases. (**B**) Production of *n*-butanol in strains expressing the optimized threonine pathway (THRm) and the synergistic pathways with *CimA* from different species. Error bars indicate standard deviations of three biological replicates. A student’s t-test was used for statistical analysis of the *n*-butanol production in triplicates. Solid pentagram (★) indicates a result that was significantly different compared to the THRm strain (*p* < 0.05).

Although the key intermediate in the endogenous *n*-butanol pathway, α-ketobutyrate, can be synthesized from the catabolism of threonine, an alternative route to α-ketobutyrate from pyruvate and acetyl coenzyme A (acetyl-CoA) via citramalate synthase (CimA) has been reported (Fig. 3A)^21^. The maximum theoretical yield of *n*-butanol is also 411 mg/g glucose via citramalate synthase. To investigate whether this alternative route could improve *n*-butanol production, candidate *CimA* genes from three distinct species, *Methanococcus jannaschii, Leptospira interrogans*, and *Geobacter sulfurreducens* that were reported previously^34^ were cloned and overexpressed with previously confirmed α-ketobutyrate utilizing genes (m*LEU1*, m*LEU4*, m*LEU2*, and *LEU5*). As shown in Fig. 3B, the highest improvement was achieved in the strain LI overexpressing *CimA* from *L. interrogans*, reaching a production of 349 mg/L of *n*-butanol, a much higher *n*-butanol production level than the strain THRm solely overexpressing the optimized threonine pathway. In strain LI, it is noteworthy that the threonine pathway still exists, thus synthesis of *n*-butanol is achieved via two metabolic pathways: the endogenous threonine pathway and the introduced citramalate pathway. The maximum theoretical yield of *n*-butanol is still 411 mg/g glucose via the synergistic pathway.

### Optimization of the synergistic pathway to improve *n*-butanol production

**Figure 4:**
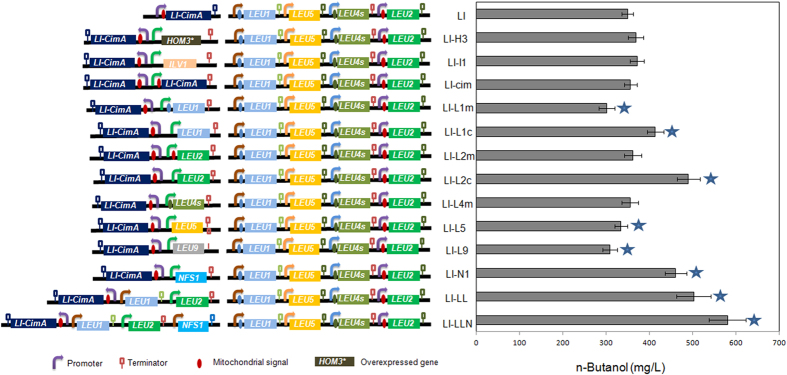
*n*-Butanol production in metabolically engineered strains by individually overexpressing an additional copy of involved genes in the synergistic pathway. All recombinant strains were constructed in the LI strain. HOM3*: *HOM3* mutant encoding *HOM3*^G1355A^ 27. *LEU4s*: truncated *LEU4* that starts at +91 ATG^33^. Fermentation was performed in anaerobic glass tubes in YPAD media with 20 g/L glucose under micro-anaerobic condition as previously described^18^. Error bars indicate standard deviations of three biological replicates. A student’s t-test was used for statistical analysis of the *n*-butanol production in triplicates. Solid pentagram (★) indicates a result that was significantly different compared to the LI strain (*p* < 0.05).

We attempted to further increase the *n*-butanol production in LI via the identified synergistic pathway (the endogenous threonine pathway and the introduced citramalate pathway) by individually overexpressing an additional copy of involved genes in the *n*-butanol producer, strain LI harboring the CimA mediated pathway. To do so, relevant genes which were previously identified (HOM3*, *ILV1*, *LEU1*, *LEU4*, *LEU2*, *LEU5*, and *CimA*), as well as *LEU9* and *NFS1* genes were individually overexpressed in the LI strain under the control of a strong constitutive promoter, P_GPD1_. As *LEU1* and *LEU2* function both in cytoplasm and mitochondria in strain LI (Fig. 3A), these two genes were both separately expressed in cytoplasm and mitochondria. *LEU9*, which encodes the same enzyme as *LEU4*, was also overexpressed. We noticed that Leu1p is an iron-sulfur (Fe-S) protein that is very sensitive to degradation^35^, while Nfs1p represents the yeast orthologue of the bacterial cysteine desulfurase NifS that initiates biogenesis by producing elemental sulfur. The matrix-localized NifS protein is required for synthesis of both mitochondrial and cytosolic Fe/S proteins^36^. Thus, *NFS1* was also overexpressed to stabilize Leu1p. As shown in Fig. 4, the highest *n*-butanol production of 490 mg/L was achieved by additional overexpression of *LEU2* in cytoplasm (strain LI-L2c). Moreover, improved *n*-butanol production was also achieved in strains overexpressing *LEU1* in cytoplasm (strain LI-L1c) and *NFS1* (strain LI-N1). Therefore, *LEU2*, *LEU1*, and *NFS1* were overexpressed together in strain LI to yield strain LI-LLN, which increased *n*-butanol production to a final titre of 580 mg/L.

### Overexpression of KDC and alcohol dehydrogenase (ADH)

**Figure 5:**
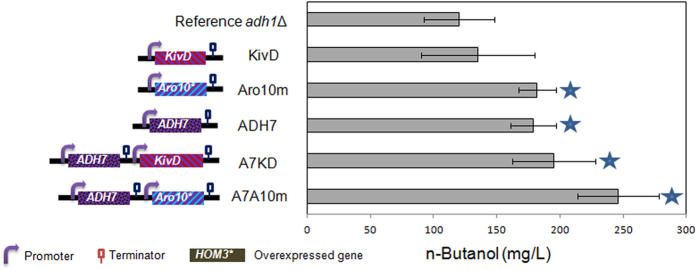
Effects of KDC and ADH overexpression on *n*-butanol production. ARO10*: *ARO10* mutant encoding Aro10p^I355Y ^46. Fermentation was performed in anaerobic glass tubes in YPAD media with 20 g/L glucose under micro-anaerobic condition as previously described^18^. Error bars indicate standard deviations of three biological replicates. A student’s t-test was used for statistical analysis of the *n*-butanol production in triplicates. Solid pentagram (★) indicates a result that was significantly different compared to the reference *adh1*Δ strain (*p* < 0.05).

In the last step of *n*-butanol biosynthesis, the reduction of α-ketovalerate to *n*-butanol can be catalyzed by KDC-like and ADH enzymes. With regards to KDCs, it has been reported that overexpression of *KivD* or a mutant *ARO10* led to improved *n*-butanol production^18^, due to their improved activity and preference toward α-ketovalerate. Concerning ADHs, in our previous study, six ADHs from *S. cerevisiae* (Adh1p–Adh6p) together with *BdhB* from *Clostridium acetobutylicum* were over-expressed in the *adh1*Δ background, and overexpression of these ADHs exhibited no accumulation or even consumption of n-butanol^18^. However, overexpression of *ADH7* could enhance isobutanol production significantly^30^. Therefore, we investigated the effect of overexpression of *ADH7* and its co-overexpression with ARO10* or *KivD*. As expected and consistent with previous studies, an enhanced *n*-butanol production was achieved in the *adh1*Δ strain. Compared to the *adh1*Δ background strain, the strains overexpressing *KivD*, ARO10*, and *ADH7* produced 12.5%, 47%, and 45% more *n*-butanol, respectively (Fig. 5). When these strategies were combined, the highest *n*-butanol producer was generated by co-overexpression of *ADH7* and ARO10*, leading to a production of 246 mg/L *n*-butanol (Fig. 5), which was 105% higher than the *adh1*Δ background strain.

### Integration of various metabolic engineering strategies

**Figure 6:**
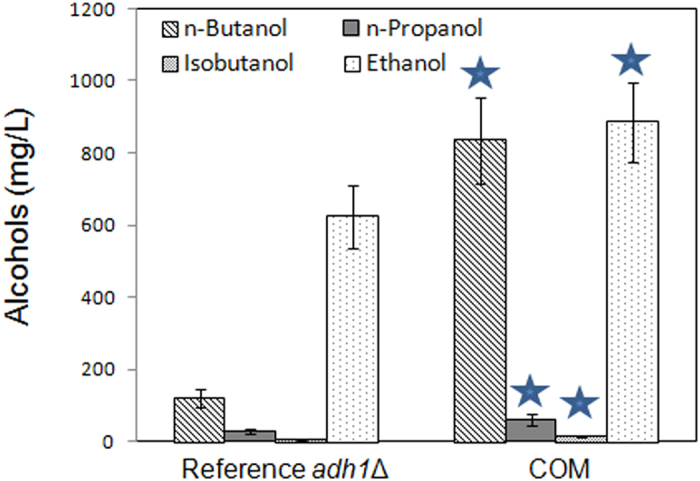
Alcohols production in strain COM and reference strain *adh1*Δ. Fermentation was performed in anaerobic glass tubes in YPAD media with 20 g/L glucose under micro-anaerobic condition as previously described^18^. Error bars indicate standard deviations of three biological replicates. A student’s t-test was used for statistical analysis of the alcohols production in triplicates. Solid pentagram (★) indicates a result that was significantly different compared to the reference *adh1*Δ strain (*p* < 0.05).

After investigating the effects of various genes related to *n*-butanol production, we sought to combine the effective strategies. As shown above, the optimized synergistic pathway performed best in *n*-butanol production, and, when we see downstream, co-overexpression of *ADH7* and ARO10* improved *n*-butanol production. After combining these two strategies, the final strain COM could produce 835 mg/L *n*-butanol in anaerobic glass tubes by the combined expression of *LEU1* (two copies), *LEU4*, *LEU2* (two copies), *LEU5, CimA, NFS1, ADH7* and ARO10* (Fig. 6), which is the highest production of *n*-butanol ever reported in yeast. The by-products were also detected in the strain COM (Fig. 6). Compared to the initial strain, there were slight increases in the production of propanol, isobutanol, and ethanol. When cultivated in a bioreactor, the strain COM could produce up to 1.05 g/L *n*-butanol with a final yield of 52.5 mg/g glucose (Supplementary Fig. S2).

## Discussion

In *S. cerevisiae*, there are two distinct pathways (CoA-dependent and keto-acid pathways) to synthesize *n*-butanol, with the keto-acid pathway showing more promising results^7^. We previously demonstrated that the endogenous *n*-butanol production was dependent on the catabolism of threonine in a manner similar to fusel alcohol production by the keto-acid pathway^18^. It has been reported that both the threonine pathway and the citramalate pathway are part of the keto-acid pathway network involved in amino acid biosynthesis^19^,^20^,^21^. In this study, several metabolic engineering strategies were applied to optimize the keto-acid pathway for *n*-butanol synthesis by modifying the threonine pathway and the citramalate pathway.

Since the endogenous *n*-butanol pathway has been discovered via threonine catabolism^18^, it would be straightforward to overexpress those genes involved in the threonine biosynthetic pathway (i.e. *HOM3, HOM2, HOM6, THR1*, and *THR4*) to supply more precursors for *n*-butanol production. However, there was no significant improvement in *n*-butanol production by overexpression of the wild-type threonine biosynthesis pathway in strain THR5. Interestingly, the sole overexpression of a *HOM3* mutant that was insensitive to feedback inhibition led to a moderate but significant increase in titer of accumulated *n*-butanol (Fig. 2). This indicated that the aspartate kinase (Hom3p) is a key enzyme in the regulation of the biosynthesis of threonine and threonine-derived products in yeast. Furthermore, co-overexpression of the *HOM3* mutant with other four genes involved in threonine biosynthetic pathway (i.e. *HOM2, HOM6, THR1*, and *THR4*) did not increase the *n*-butanol production compared to strain Hom3m (Fig. 2). This was consistent with previous results showing that threonine biosynthesis is tightly regulated and only the amplification of the mutant *HOM3* allele can enable deregulation and overproduction of threonine^26^,^27^,^37^. On the other hand, Ilv1p, Leu4p, Leu1p, and Leu2p catalyze a series of reactions that convert threonine to α-ketovalerate (Fig. 1A), and all of them play a role in stimulating *n*-butanol production^18^. Therefore, it was not surprising that co-overexpression of these genes further improved *n*-butanol production (Fig. 2).

Relocalization of pathway genes in different compartments is an effective metabolic engineering strategy. For isobutanol synthesis, relocalization of the valine biosynthesis and degradation pathway into either cytosol or mitochondria led to improved isobutanol production^29^,^30^. However, the mitochondrial membrane can enable the separation of α-ketobutyrate and cytosolic KDC-like and ADH enzymes to avoid or decrease the production of byproduct propanol^18^. Accordingly, only low amount of *n*-propanol is detected in the *adh1*Δ strains (Fig. 6), which is an advantage in contrast to the unavoidable production of *n*-propanol in *E. coli*^19^. Therefore, KDC-like and ADH enzymes were not relocalized into mitochondria in strain THRm in this study. It was found that the relocalization of *LEU* genes mattered to the threonine pathway based *n*-butanol production (Fig. 2); strain THRm with relocalized-enzymes in mitochondria produced more *n*-butanol compared to the corresponding strain THRc (Fig. 2). This is probably because the smaller volume of mitochondria could concentrate threonine, resulting in faster reaction rates. The different environment in the mitochondrial matrix may also help the function of certain enzymes.

In addition to the threonine biosynthesis based pathway, *n*-butanol production could also be achieved via the CimA mediated pathway^20^,^21^ (Fig. 3A). Here, three candidate *CimA* genes from different distinct species were overexpressed with previously confirmed α-ketobutyrate utilizing genes (m*LEU1*, m*LEU4*, m*LEU2*, and *LEU5*). The highest improvement was achieved in the strain LI overexpressing *CimA* from *L. interrogans*, reaching a production of 349 mg/L of *n*-butanol (Fig. 3B). When the citramalate pathway was heterogeneously expressed, since the endogenous threonine pathway is still there and may act to contribute to the final *n*-butanol titer, the formed *n*-butanol is produced via a synergistic pathway: the endogenous threonine pathway and the introduced citramalate pathway. This phenomenon was also observed in *E. coli*^20^,^21^. However, as there is no mitochondrion in *E. coli* to separate α-ketobutyrate and KDC/ADH enzymes, a large amount of propanol was formed, making it a less desirable *n*-butanol production host compared to yeast, in which little or low propanol is formed^18^ (Fig. 6). Further optimization was achieved by overexpression of all relevant genes in the CimA mediated synergistic pathway. Notably, the improvement of *n*-butanol production was achieved in strains overexpressing *LEU1*, *LEU2* or *NFS1* in cytoplasm (Fig. 4), and all these genes contributed to a higher conversion rate of 2-ethyl-malate to α-ketovalerate in cytoplasm, in parallel to the corresponding conversion happened in mitochondrion. This suggests that an orthogonal pathway to synthesize α-ketovalerate gave an additional improvement in *n*-butanol production.

The subsequent decarboxylation and reduction of α-ketovalerate to *n*-butanol is catalyzed by KDC-like and ADH enzymes. There are no specific wild-type KDCs or ADHs enzymes to catalyze this step. We found that the co-overexpression of *ADH7* and ARO10* (strain A7A10m) or *ADH7* and Kivd (strain A7KD) were both successful strategies to overproduce *n*-butanol (Fig. 5), and the co-overexpression of *ADH7* and ARO10* gave a better results (246 mg/L), indicating that ARO10* encoded KDC may have a higher activity for α-ketovalerate to form n-butanol. On the other hand, overexpressed *ARO10* could also be used as a metabolic engineering approach to enhance the production of 2-phenylethanol via the keto-acid pathway^38^,^39^, suggesting that Aro10p might play an important role in aromatic amino acid reduction. ARO10* was also identified to encode a phenylpyruvate decarboxylase with a broad spectrum of activity, and enabled the accumulation of different fusel alcohols^40^. The expression of *ADH7* was reported to have a positive effect on the isobutanol production^30^, and we showed its positive effect on the *n*-butanol production for the first time. Moreover, Adh7p is a promiscuous enzyme with broad substrate specificity, and participates in the synthesis of fusel alcohols^41^. Although the co-overexpression of *ADH7* and ARO10* facilitated the production of *n*-butanol, it is still highly desirable to engineer or select KDCs/ADHs with higher preference toward α-ketovalerate/butyraldehyde, which could decrease the formation of side products such as ethanol (Fig. 6). Pyruvate is the precursor of the CimA mediated pathway. Because its ethanol production was completely abolished and pyruvate was accumulated to a high level, the previously engineered *pdc*- strain^11^ can serve as a good starting point for the construction a new *n*-butanol producer.

Our final engineered yeast strain COM resulted in the highest *n*-butanol production at 835 mg/L in a anaerobic glass tube, which is nearly 3-fold of that previously reported highest *n*-butanol yeast producer (300 mg/L)^25^. Low level of *n*-propanol and isobutanol is observed in strain COM (Fig. 6), however the production of ethanol is still high, indicating a necessity to abolish its production. Our metabolic engineering strategies were implemented via multiple plasmids and we believe that chromosomal integration may further improve the *n*-butanol production because the strain was cultured in YPAD medium without any selective pressure to maintain the plasmids. In addition, note that the final strain COM can produce up to 1.05 g/L *n*-butanol in a bioreactor. However, the performance was not stable possibly because the oxygen level was too low to be precisely detected and controlled by the dissolved oxygen probe. It has been also indicated that a small amount of oxygen was necessary to achieve higher *n*-butanol productivity^42^. It is thought the fluctuation in dissolved oxygen affected the yeast physiology, thus further optimization in the process control of dissolved oxygen is required to enhance the *n*-butanol production.

## Conclusion

In this work we evaluated and optimized a synergistic pathway (the endogenous threonine pathway and the introduced citramalate pathway) to supply precursor α-ketovalerate for *n*-butanol production. Then, overexpression of KDC-like and ADH enzymes were also used to push the flux from α-ketovalerate to *n*-butanol. The combination of these metabolic engineering strategies increased the production of *n*-butanol from 120 mg/L to 835 mg/L in anaerobic glass tubes, a 7-fold improvement. This is the highest reported *n*-butanol titer in *S. cerevisiae* with a yield of 42 mg/g glucose. As a case study, utilization of such a synergistic pathway improves versatility of production condition and highlights the significance of synergy in pathway design and construction.

## Methods

### DNA manipulation and plasmids

All DNA manipulations were carried out in *E. coli* DH5α as described^43^. Plasmid cloning was performed using Gibson Assembly Cloning Kit (New England Biolabs, UK) following the manufacturer’s instructions. Briefly, DNA fragments sharing homologous regions to adjacent DNA fragments were generated via polymerase chain reaction (PCR) or restriction enzymes digestions. The desired DNA fragments were purified with QIAquick Gel Extraction Kit (Qiagen, Valencia, CA) and assembled with the linearized backbone in a single step. The sequences of all PCR primers and templates used are listed in Supplementary Table S1 in the supplemental material. Primers were synthesized by Sigma Aldrich (Singapore). The complete list of fragments for plasmid construction by DNA assembly in this study is summarized in Supplementary Table S1. QIAprep Spin Plasmid Mini-prep Kits (Qiagen, Valencia, CA) were employed to prepare plasmid DNA from *E. coli*. All restriction enzymes were obtained from New England Biolabs.

### Strains

*S. cerevisiae* strain YSG50 *ura3*Δ (*MATα ura3*Δ *ade2-1 his3-11,15 leu2-3,112 can1-100 trp1-1*) was designated as the wild-type (WT) strain, and its *ADH1* deletion strain, the *adh1*Δ strain, was adopted as the reference *n*-butanol producer^18^. The complete list of strains in this study is summarized in Supplementary Table S2. The method used for yeast transformation is the standard LiAc/SS carrier DNA/PEG method^44^.

### Media and growth conditions

*E. coli* recombinant cells were grown in Luria-Bertani (LB) medium in the presence of ampicillin (100 mg/L) at 37 °C and 250 rpm. *S. cerevisiae* strains were grown on synthetic dropout (SD) medium agar plates containing the appropriate medium composition for selection (0.17% Difco yeast nitrogen base without amino acids and ammonium sulfate, 0.5% ammonium sulfate and 0.083% amino acid dropout mix, 0.01% adenine hemisulfate and 2% glucose).

For *n*-butanol production in a shake-flask, *S. cerevisiae* strains were cultivated in YPAD medium (1% yeast extract, 2% peptone, 0.01% adenine hemisulfate and 2% glucose) at 30 °C with 250 rpm agitation in baffled shake-flasks for aerobic growth. For micro-anaerobic fermentation, 4 mL cultures of resting cells were grown in Bellco 18 × 150 mm anaerobic glass tubes sealed with rubber stoppers and aluminium crimps (Chemglass, Vineland, NJ). The initial OD of the cultures was around 1.0 after inoculation from pre-cultures. Vacuum was applied through a syringe needle for 20 minutes, and sterile nitrogen was then added to create micro-aerobic conditions.

For *n*-butanol production in a bioreactor, micro-anaerobic batch fermentation of resting cells was performed in YPAD medium with 20 g/L glucose in 1.0 L Dasgip stirrer-pro® bioreactors (DasGip, Jülich, Germany) with 0.24 L working volume. The cultivation was carried out with an agitation of 400 rpm and at a controlled temperature of 30 °C without any aeration. Dissolved oxygen was measured using an O_2_ sensor (Mettler Toledo, Columbus, OH). The initial OD of the cultures was around 1.0 after inoculation from pre-cultures.

### Analytical methods

Samples were collected after 96 hours of cultivation, and centrifuged at 12,000 rpm for 5 min. The supernatant was filtered and stored at −80 °C prior to analysis to minimize evaporation. The concentration of *n*-butanol in the filtered supernatant was determined by gas chromatography-mass spectroscopy using a Shimadzu GC-MS QP 2010 (Shimadzu Corporation, Kyoto, Japan) and HP-INNOWAX column (Agilent Inc., Palo Alto, CA). Helium was used as the carrier gas. Mass transfer line and ion source were held at 250 °C and 230 °C, respectively. The oven temperature program was set as in our previous study^18^. The serial dilutions of *n*-butanol standards were injected in the same analysis to generate standard curves for *n*-butanol quantification performed with help of the quantitative function in Shimadzu GCMSsolutions Software Ver. 2.6 (Shimadzu Corporation Kyoto, Japan).

### Statistical analysis

All experiments were conducted in biological triplicates unless otherwise stated. Student’s t-test was performed to estimate whether the influence of a variable is significant. A value of p < 0.05 was considered statistically significant.

## Additional Information

**How to cite this article**: Shi, S. *et al*. Metabolic engineering of a synergistic pathway for *n*-butanol production in *Saccharomyces cerevisiae*. *Sci. Rep*. **6**, 25675; doi: 10.1038/srep25675 (2016).

## Supplementary Material

Supplementary Information
