# Supplementary material for: Metabolic engineering of a synergistic pathway for n-butanol production in Saccharomyces cerevisiae
Source: Sci Rep. 2016 May 10;6:25675. doi: 10.1038/srep25675 (PMC4861978; doi:10.1038/srep25675)
Supplement: Supplementary Information [file srep25675-s1.doc]

# Supplementary Information

**Metabolic engineering of a synergistic pathway for n-butanol production in *Saccharomyces cerevisiae***

Shuobo Shi^1^, Tong Si^2^, Zihe Liu^1^, Hongfang Zhang^1^, Ee Lui Ang^1, *^, Huimin Zhao^1,2, *^

^1^Metabolic Engineering Research Laboratory, Science and Engineering Institutes, Agency for Science, Technology and Research, Singapore

^2^Department of Chemical and Biomolecular Engineering, University of Illinois at Urbana-Champaign, Urbana, IL 61801

^*^ To whom correspondence should be addressed.

Huimin Zhao: Phone: (217) 333-2631. Fax: (217) 333-5052. E-mail: zhao5@illinois.edu

Ee Lui Ang: Phone: (+65) 6419 6650. E-mail: angel@merl.a-star.edu.sg

**Supplementary Table S1**. List of primers and templates used for plasmids construction. All plasmids are assembled by Gibson assembly cloning Kit (New England Biolabs, MA, USA) following the manufacturer’s instructions.

| **Plasmid** | **Description** | **Fragments for plasmid construction by assembling** | **PCR Template** | | **Primer sequence** |
| --- | --- | --- | --- | --- | --- |
| pRS423 | 2μ *ori*, HIS3 marker | Adopted from previous work^1^ | | | |
| pRS424 | 2μ *ori*, TRP1 marker | Adopted from previous work^1^ | | | |
| pRS426 | 2μ *ori*, URA3 marker | Adopted from previous work^1^ | | | |
| pRS426-THR | P_TEF1_-*HOM3*-T_PGK1_-P_TPI1_-*HOM2*-T_GPD1_-P_ENO2_-*HOM6*-T_TEF1_-P_PDC1_-*THR1*-T_HXT7_-P_FBA1_-*THR4*-T_TEF2_ | Adopted from previous work^2^ | | | |
| pSS01  Continued on next page | P_TEF1_-GFP-T_ADH1_ | *Kpn*I/*Sac*I-digested pRS426 | - | - | |
|  |  | P_TEF1_ | Genomic DNA of *S. cerevisiae* HZ848 | cgcgcgtaatacgactcactatagggcgaattgggtaccagcaacaggcgcgttggac | |
|  |  |  |  | ggacaactccagtgaaaagttcttctcctttactcattttgtaattaaaacttagattag | |
|  |  | GFP | Plasmid pRS426-PDC1p-GFP-PDC1t^3^ | gaaagcatagcaatctaatctaagttttaattacaaaatgagtaaaggagaagaac | |
|  |  |  |  | cataaatcataagaaattcgctatttgtatagttcatccatg | |
|  |  | T_ADH1_ | Genomic DNA of *S. cerevisiae* HZ848 | catggatgaactatacaaatagcgaatttcttatgatttatg | |
|  |  |  |  | ccctcactaaagggaacaaaagctggagctcgagcgacctcatgctatacc | |
| **Plasmid** | **Description** | **Fragments for plasmid construction by assembling** | **PCR Template** | **Primer sequence** | |
| pSS02 | P_TEF1_-CoxIVm-GFP-T_ADH1_ | pRS426-P_TEF1_-CoxIVm-GFP- T_ADH1_ | Plasmid pSS01 | taagatttttcaagccagccacaagaactttgt gtagctctagatatctgcttagtaaaggagaagaacttttca | |
|  |  |  |  | gtggctggcttgaaaaatcttatagattgacgtagtgaaagcattttgtaattaaaacttagattag | |
| pSS03 | P_TEF1_-CYB2m-GFP-T_ADH1_ | pRS426-P_TEF1_-CYB2m-GFP- T_ADH1_ | Plasmid pSS01 | tcctcagagcgtctaagactagattgaacacaatccgcgcgtacggttctaccgttccaaaatccagtaaaggagaagaacttttc | |
|  |  |  |  | gtcttagacgctctgaggatagcagcctcacagttcttcgagatttttagtaaaggtttgtattttagcatttttgtaattaaaacttag | |
| pSS04 | P_TEF1_-CAT2m-GFP-T_ADH1_ | pRS426-P_TEF1_-CAT2m-GFP- T_ADH1_ | Plasmid pSS01 | ctctctcaaacttaaaggatcttccgataacgtcaaggagagcaagtaaaggagaagaacttttc | |
|  |  |  |  | atcctttaagtttgagagagttctcgaatgacagatcctcattttgtaattaaaacttaga | |
| pRS426-HOM3* | P_TEF1_-*HOM3**-T_ADH1_ (*HOM3** encodes *HOM3*^G1355A^ ^4^) | pRS426-P_TEF1_-T_ADH1_ | Plasmid pSS01 | gtttagaacaattgaaaagacttggaatttaacgaatttcttatgatttatg | |
|  |  |  |  | gacttgatgtaggttggaaatccattggcatttttgtaattaaaacttagattag | |
|  |  | *HOM3-1* | Genomic DNA of *S. cerevisiae* HZ848 | tacaaaaatgccaatggatttccaacc | |
|  |  |  |  | ggtaccagcaatgtcgatgtattgtttcatatg | |
|  |  | *HOM3-2* | Genomic DNA of *S. cerevisiae* HZ848 | gaaacaatacatcgacattgctggtaccatgtttac | |
|  |  |  |  | gaaattcgttaaattccaagtcttttcaattg | |
| pRS426-THR* | P_TEF1_-*HOM3**-T_ADH1_- P_TPI1_-*HOM2*-T_GPD1_-P_ENO2_-*HOM6*-T_TEF1_-P_PDC1_-*THR1*-T_HXT7_-P_FBA1_-*THR4*-T_TEF2_ | *Sac*I-digested pRS426-P_TEF1_-*HOM3**-T_ADH1_ | - | - | |
|  |  | P_TPI1_-*HOM2*-T_GPD1_-P_ENO2_-*HOM6*-T_TEF1_ | Plasmid pRS426-THR | gtcaggttgctttctcaggtatagcatgaggtcgctctatatctaggaacccatcag | |
|  |  |  |  | cagtcgcatgatagcgccga | |
|  |  | P_PDC1_-*THR1*-T_HXT7_-P_FBA1_-*THR4*-T_TEF2_ | Plasmid pRS426-THR | actttgatcggcgctatc | |
|  |  |  |  | caattaaccctcactaaagggaacaaaagctggagctcggggtagcgacggattaatg | |
| Continued on next page | | | | | |
|  | | | | | |
|  | | | | | |
| **Plasmid** | **Description** | **Fragments for plasmid construction by assembling** | **PCR Template** | **Primer sequence** | |
| pRS424-LEU1 | P_PGK1_-*LEU1*-T_GPD1_ | *Kpn*I/*Sac*I-digested pRS424 | - | - | |
|  |  | P_PGK1_ | Genomic DNA of *S. cerevisiae* HZ848 | cgactcactatagggcgaattgggtaccggaagtaccttcaaagaatg | |
|  |  |  |  | gagtgtaaaccattgttttatatttgttgt | |
|  |  | *LEU1* | Genomic DNA of *S. cerevisiae* HZ848 | acaacaaatataaaacaatggtttacactccatcc | |
|  |  |  |  | gatttaaagtaaattcacctaccaatcctggtggac | |
|  |  | T_GPD1_ | Genomic DNA of *S. cerevisiae* HZ848 | caccaggattggtaggtgaatttactttaaatc | |
|  |  |  |  | cctcactaaagggaacaaaagctggagctcggaatctgtgtatattac | |
| pRS424-LEU5 | P_TPI1_-*LEU5*-T_HXT7_ | *Kpn*I/*Sac*I-digested pRS424 | - | - | |
|  |  | P_TPI1_ | Genomic DNA of *S. cerevisiae* HZ848 | acgactcactatagggcgaattgggtacctatatctaggaacccatcag | |
|  |  |  |  | ctatctcgcgtcatttttagtttatgtatgtg | |
|  |  | *LEU5* | Genomic DNA of *S. cerevisiae* HZ848 | cacatacataaactaaaaatgacgcgagatagcccag | |
|  |  |  |  | catgaattaataaaagtgttcgcaaattaaatgccaaaattcc | |
|  |  | T_HXT7_ | Genomic DNA of *S. cerevisiae* HZ848 | ggaattttggcatttaatttgcgaacacttttattaattcatg | |
|  |  |  |  | cactaaagggaacaaaagctggagctcataactgactcattagacac | |
| pRS424-LEU4 | P_GPM1_-*LEU4*-T_PGK1_- | *Kpn*I/*Sac*I-digested pRS424 | - | - | |
|  |  | P_GPM1_ | Genomic DNA of *S. cerevisiae* HZ848 | ctctctttaaccattattgtaatatgtgtgtttgtttgg | |
|  |  |  |  | ccctcactaaagggaacaaaagctggagctctagtcgtgcaatgtatgac | |
|  |  | *LEU4*-T_PGK1_ | Plasmid pRS425-LEU^2^ | cgcgcgtaatacgactcactatagggcgaattgggtacc caggaagaatacactatac | |
|  |  |  |  | caaacaaacacacatattacaataatggttaaagagagtattattgc | |
| Continued on next page | | | | | |
|  | | | | | |
| **Plasmid** | **Description** | **Fragments for plasmid construction by assembling** | **PCR Template** | **Primer sequence** | |
| pRS424-LEU2 | P_ENO2_-*LEU2*-T_PGI1_ | *Kpn*I/*Sac*I-digested pRS424 | - | - | |
|  |  | P_ENO2_ | Genomic DNA of *S. cerevisiae* HZ848 | cttcttaggggcagacatttattattgtatgttatag | |
|  |  |  |  | ccctcactaaagggaacaaaagctggagctcgtgtcgacgctgcgggtatag | |
|  |  | *LEU2* | Genomic DNA of *S. cerevisiae* HZ848 | ggtatatatttaagagcgatttgtttaagcaaggattttcttaac | |
|  |  |  |  | ctataacatacaataataaatgtctgcccctaagaagatc | |
|  |  | T_PGI1_ | Genomic DNA of *S. cerevisiae* HZ848 | gtaatacgactcactatagggcgaattgggtaccggtatactggaggcttcatg | |
|  |  |  |  | gttaagaaaatccttgcttaaacaaatcgctcttaaatatatacc | |
| pRS424-LEU | P_PGK1_-*LEU1*-T_GPD1_-P_TPI1_-*LEU5*-T_HXT7_-P_GPM1_-*LEU4*-T_PGK1_-P_ENO2_-*LEU2*-T_PGI1_ | *Kpn*I/*Sac*I-digested pRS424 | - | - | |
|  |  | P_PGK1_-*LEU1*-T_GPD1_ | Plasmid pRS424-LEU1 | cgactcactatagggcgaattg | |
|  |  |  |  | caacctgatgggttcctagatataggaatctgtgtatattac | |
|  |  | P_TPI1_-*LEU5*-T_HXT7_ | Plasmid pRS424-LEU5 | cagtaatatacacagattcctatatctaggaacccatcagg | |
|  |  |  |  | agtgtattcttcctgataactgactcattagacac | |
|  |  | P_GPM1_-*LEU4*-T_PGK1_ | Plasmid pRS424-LEU4 | gtctaatgagtcagttatcaggaagaatacactatac | |
|  |  |  |  | catgaagcctccagtatacctagtcgtgcaatgtatgac | |
|  |  | P_ENO2_-*LEU2*-T_PGI1_ | Plasmid pRS424-LEU2 | gtcatacattgcacgactaggtatactggaggcttcatg | |
|  |  |  |  | ccctcactaaagggaacaaaagctggagctcgtgtcgacgctgcgggtatag | |
| pRS424-mLEU1 | P_PGK1_-CoxIVm-*LEU1*-T_GPD1_ | pRS424-P_PGK1_-CoxIVm-*LEU1*-T_GPD1_ | Plasmid pRS424-LEU1 | gatttttcaagccagccacaagaactttgtgtagctctagatatctgcttgtttacactccatccaag | |
|  |  |  |  | gttcttgtggctggcttgaaaaatcttatagattgacgtagtgaaagcattgttttatatttgttgta | |
| pRS424-mLEU4s  Continued on next page | P_GPM1_-CAT2m-*LEU4s*-T_PGK1_ (The short version *LEU4*, *LEU4s*, is started from +91 ATG of original *LEU4*^5^) | pRS424-P_GPM1_-CAT2m-*LEU4s*-T_PGK1_ | Plasmid pRS424-LEU4 | ctttaagtttgagagagttctcgaatgacagatcctcattattgtaatatgtgtgtttg | |
|  |  |  |  | Tctctcaaacttaaaggatcttccgataacgtcaaggagagcacttaaggacccttcctccaag | |
| **Plasmid** | **Description** | **Fragments for plasmid construction by assembling** | **PCR Template** | **Primer sequence** | |
| pRS424-mLEU2 | P_ENO21_-CYB2m-*LEU2*-T_PGI1_ | pRS424-P_ENO21_-CYB2m-*LEU2*-T_PGI1_ | Plasmid pRS424-LEU2 | tagacgctctgaggatagcagcctcacagttcttcgagatttttagtaaaggtttgtattttagcatttattattgtatgttatag | |
|  |  |  |  | ctatcctcagagcgtctaagactagattgaacacaatccgcgcgtacggttctaccgttccaaaatcctctgcccctaagaagatcg | |
| pRS424-mLEU | P_PGK1_-CoxIVm-*LEU1*-T_GPD1_-P_TPI1_-LEU5-T_HXT7_-P_GPM1_- CAT2m-*LEU4s*-T_PGK1_- P_ENO21_-CYB2m-*LEU2*-T_PGI1_ | *Kpn*I/*Sac*I-digested pRS424 | - | - | |
|  |  | pRS424-P_PGK1_-CoxIVm-*LEU1*-T_GPD1_ | Plasmid pRS424-mLEU1 | cgactcactatagggcgaattg | |
|  |  |  |  | caacctgatgggttcctagatataggaatctgtgtatattac | |
|  |  | P_TPI1_-*LEU5*-T_HXT7_ | Plasmid pRS424-LEU5 | cagtaatatacacagattcctatatctaggaacccatcagg | |
|  |  |  |  | agtgtattcttcctgataactgactcattagacac | |
|  |  | pRS424-P_GPM1_-CAT2m-*LEU4s*-T_PGK1_ | Plasmid pRS424-mLEU4s | gtctaatgagtcagttatcaggaagaatacactatac | |
|  |  |  |  | catgaagcctccagtatacctagtcgtgcaatgtatgac | |
|  |  | pRS424-P_ENO21_-CYB2m-*LEU2*-T_PGI1_ | Plasmid pRS424-mLEU2 | gtcatacattgcacgactaggtatactggaggcttcatg | |
|  |  |  |  | ccctcactaaagggaacaaaagctggagctcgtgtcgacgctgcgggtatag | |
| pRS426- MJ_CimA | P_TEF1_-MJ_CimA-T_ADH1_ (MJ_CimA, *CimA* from *Methanococcus jannaschii*) | pRS426-P_TEF1_-T_ADH1_ | Plasmid pSS01 | ggaaggaatcaatatgttattgaattaacgaatttcttatgatttatg | |
|  |  |  |  | atcaaatatccttaccatcatttttgtaattaaaacttagattag | |
|  |  | MJ_*CimA* | Genomic DNA of *M. jannaschii* [DSM-2661](http://www.dsmz.de/catalogues/details/culture/DSM-2661.html?tx_dsmzresources_pi5%5BreturnPid%5D=304) (purchased from DSMZ, Deutsche Sammlung von Mikroorganismen und Zellkulturen ) | ctaagttttaattacaaaaatgatggtaaggatatttg | |
|  |  |  |  | cataaatcataagaaattcgttaattcaataacatattgattcct | |
| pRS426- LI_CimA  Continued on next page | P_TEF1_-LI_CimA-T_ADH1_ (LI_CimA, *CimA* from *Leptospira interrogans*) | pRS426-P_TEF1_-T_ADH1_ | Plasmid pSS01 | caatcaaattctacaaccatggcaaatctaacgaatttcttatgatttatg | |
|  |  |  |  | caatcgagtttctacttttgtcatttttgtaattaaaacttagattag | |
|  |  | LI_*CimA* | Genomic DNA of *L.interrogans* DSM-21525 (purchased from DSMZ) | ctaagttttaattacaaaaatgacaaaagtagaaactcgattgg | |
|  |  |  |  | cataaatcataagaaattcgttagatttgccatggttgtag | |
| **Plasmid** | **Description** | **Fragments for plasmid construction by assembling** | **PCR Template** | **Primer sequence** | |
| pRS426- GS_CimA | P_TEF1_-GS_CimA-T_ADH1_ (GS_CimA, *CimA* from *Geobacter sulfurreducens*) | pRS426-P_TEF1_-T_ADH1_ | Plasmid pSS01 | caagaagtgaaaccccgcaccgcgaatttcttatgatttatg | |
|  |  |  |  | gtatcgtagagttttacaaggctcatttttgtaattaaaacttagattag | |
|  |  | GS_*CimA* | Genomic DNA of *G.sulfurreducens* DSM-12127 (purchased from DSMZ) | gttttaattacaaaaatgagccttgtaaaactctacg | |
|  |  |  |  | cataaatcataagaaattcgcggtgcggggtttcacttctt | |
| pRS424-cLEU | P_PGK1_-*LEU1*-T_GPD1_-P_TPI1_-*LEU5*-T_HXT7_-P_GPM1_-*LEU4s*-T_PGK1_-P_ENO2_-*LEU2*-T_PGI1_ | pRS424- P_PGK1_-*LEU1*-T_GPD1_-P_TPI1_-*LEU5*-T_HXT7_-P_GPM1_-*LEU4s*-T_PGK1_-P_ENO2_-*LEU2*-T_PGI1_ | pRS424-LEU | tggaggaagggtccttaagcattattgtaatatgtgtgtttgtttgg | |
|  |  |  |  | ccaaacaaacacacatattacaataatgcttaaggacccttcctc | |
| pRS414- P_GDP1_-CYB2m-Li_CimA- T_PGK1_ | P_GDP1_-CYB2m-LI_*CimA*-T_PGK1_ | *Xho*I-digested pRS414-P_GDP1_-T_PGK1_ (plasmid pRS414-P_GDP1_-T_PGK1_ is from ref. 6) | - | - | |
|  |  | CYB2m-LI_*CimA* | Plasmid pRS426- CYB2m-LI_CimA | gaacttagtttcgacggatcatgctaaaatacaaacctttac | |
|  |  |  |  | cttcattcaatcatgattctttttcttagatttgccatggttgtag | |
| pRS414- P_GDP1_- HOM3*-T_PGK1_ | P_GDP1_- *HOM3**-T_PGK1_ | *Xho*I-digested pRS414-P_GDP1_-T_PGK1_ | - | - | |
|  |  | HOM3* | pRS426-HOM3* | cttagtttcgacggatcatgccaatggatttccaacc | |
|  |  |  |  | tcaatcatgattctttttcttaaattccaagtcttttcaattg | |
| pRS414- P_GDP1_- ILV1-  T_PGK1_ | P_GDP1_-*ILV1*-T_PGK1_ | *Xho*I-digested pRS414-P_GDP1_-T_PGK1_ | - | - | |
|  |  | *ILV1* | Genomic DNA of *S. cerevisiae* HZ848 | cttagtttcgacggatcatgtcagctactctactaaagc | |
|  |  |  |  | tcaatcatgattctttttcttaatatttcaagaatttttg | |
| pRS414- P_GDP1_- CoXIVm-LEU1-T_PGK1_ | P_GDP1_-CoXIVm-*LEU1*-T_PGK1_  Continued on next page | *Xho*I-digested pRS414-P_GDP1_-T_PGK1_ | - | - | |
|  |  | CoXIVm-*LEU1* | Plasmid pRS424-mLEU1 | cttagtttcgacggatcatgctttcactacgtcaatc | |
|  |  |  |  | tcaatcatgattctttttcctaccaatcctggtggac | |
| **Plasmid** | **Description** | **Fragments for plasmid construction by assembling** | **PCR Template** | **Primer sequence** | |
| pRS414- P_GDP1_- LEU1-T_PGK1_ | P_GDP1_-*LEU1*-T_PGK1_ | *Xho*I-digested pRS414-P_GDP1_-T_PGK1_ | - | - | |
|  |  | *LEU1* | Plasmid pRS424-LEU1 | cttagtttcgacggatcatggtttacactccatcc | |
|  |  |  |  | tcaatcatgattctttttcctaccaatcctggtggac | |
| pRS414- P_GDP1_- CYB2m-LEU2-T_PGK1_ | P_GDP1_-CYB2m-*LEU2*-T_PGK1_ | *Xho*I-digested pRS414-P_GDP1_-T_PGK1_ | - | - | |
|  |  | CYB2m-*LEU2* | Plasmid pRS424-mLEU2 | cttagtttcgacggatcatgctaaaatacaaacctttactaaaaatc | |
|  |  |  |  | tcaatcatgattctttttcttaagcaaggattttcttaacttc | |
| pRS414- P_GDP1_- LEU2-T_PGK1_ | P_GDP1_-*LEU2*-T_PGK1_ | *Xho*I-digested pRS414-P_GDP1_-T_PGK1_ | - | - | |
|  |  | *LEU2* | Plasmid pRS424-LEU2 | cttagtttcgacggatcatgtctgcccctaagaag | |
|  |  |  |  | tcaatcatgattctttttcttaagcaaggattttcttaacttc | |
| pRS414- P_GDP1_- CAT2m-LEU4s- T_PGK1_ | P_GDP1_-CAT2m-*LEU4s*-T_PGK1_ | *Xho*I-digested pRS414-P_GDP1_-T_PGK1_ | - | - | |
|  |  | CAT2m-*LEU4s* | Plasmid pRS424-mLEU4s | cttagtttcgacggatcatgaggatctgtcattc | |
|  |  |  |  | tcaatcatgattctttttcttatgcagagccagatgcc | |
| pRS414- P_GDP1_- LEU5-T_PGK1_ | P_GDP1_-*LEU5*-T_PGK1_ | *Xho*I-digested pRS414-P_GDP1_-T_PGK1_ | - | - | |
|  |  | *LEU5* | Genomic DNA of *S. cerevisiae* HZ848 | cttagtttcgacggatcatgacgcgagatagcccag | |
|  |  |  |  | tcaatcatgattctttttcttaaatgccaaaattccatttcattctttc | |
| pRS414- P_GDP1_- LEU9-T_PGK1_ | P_GDP1_-*LEU9*-T_PGK1_ | *Xho*I-digested pRS414-P_GDP1_-T_PGK1_ | - | - | |
|  |  | LEU9 | Genomic DNA of *S. cerevisiae* HZ848 | cttagtttcgacggatcatggtaaaacattcgttc | |
|  |  |  |  | tcaatcatgattctttttcttactctgccagtagaacatc | |
| pRS414- P_GDP1_- NFS1-T_PGK1_  Continued on next page | P_GDP1_-*NFS1*-T_PGK1_ | *Xho*I-digested pRS414-P_GDP1_-T_PGK1_ | - | - | |
|  |  | *NFS1* | Genomic DNA of *S. cerevisiae* HZ848 | cttagtttcgacggatcatgttgaaatcaactgctac | |
|  |  |  |  | tcaatcatgattctttttctcaatgacctgaccatttg | |
| **Plasmid** | **Description** | **Fragments for plasmid construction by assembling** | **PCR Template** | **Primer sequence** | |
| pRS426-Li-cimA_2copy | P_TEF1_-CYB2m-LI_*CimA*-T_ADH1_-P_GDP1_-CYB2m-LI_*CimA*-T_PGK1_ | *Kpn*I-digested pRS426- CYB2m-LI_CimA | - | - | |
|  |  | P_GDP1_-CYB2m-LI_*CimA* -T_PGK1_ | Plasmid pRS414- P_GDP1_-CYB2m-Li_CimA-T_PGK1_ | gggcgaattgggtaccatttatgtacccatgtataacc | |
|  |  |  |  | cgcctgttgctggtacagtttatcattatcaatactcgc | |
| pRS426-Li-cimA-HOM3* | P_TEF1_-CYB2m-LI_*CimA*-T_ADH1_-P_GDP1_- HOM3*-T_PGK1_ | *Kpn*I-digested pRS426- CYB2m-LI_CimA | - | - | |
|  |  | P_GDP1_- HOM3*-T_PGK1_ | pRS414-P_GDP1_- HOM3*-T_PGK1_ | gggcgaattgggtaccatttatgtacccatgtataacc | |
|  |  |  |  | cgcctgttgctggtacagtttatcattatcaatactcgc | |
| pRS426-Li-cimA-ILV1 | P_TEF1_-CYB2m-LI_*CimA*-T_ADH1_-P_GDP1_- ILV1-T_PGK1_ | *Kpn*I-digested pRS426- CYB2m-LI_CimA | - | - | |
|  |  | P_GDP1_- ILV1-T_PGK1_ | pRS414-P_GDP1_-ILV1-  T_PGK1_ | gggcgaattgggtaccatttatgtacccatgtataacc | |
|  |  |  |  | cgcctgttgctggtacagtttatcattatcaatactcgc | |
| pRS426-Li-cimA-mLeu1 | P_TEF1_-CYB2m-LI_*CimA*-T_ADH1_-P_GDP1_-CoXIVm-*LEU1*- T_PGK1_ | *Kpn*I-digested pRS426- CYB2m-LI_CimA | - | - | |
|  |  | P_GDP1_-CoXIVm-*LEU1*-T_PGK1_ | Plasmid pRS414- P_GDP1_- CoXIVm-LEU1- T_PGK1_ | gggcgaattgggtaccatttatgtacccatgtataacc | |
|  |  |  |  | cgcctgttgctggtacagtttatcattatcaatactcgc | |
| pRS426-Li-cimA-Leu1 | P_TEF1_-CYB2m-LI_*CimA*-T_ADH1_-P_GDP1_-*LEU1*-T_PGK1_ | *Kpn*I-digested pRS426- CYB2m-LI_CimA | - | - | |
|  |  | P_GDP1_-*LEU1*-T_PGK1_ | Plasmid pRS414- P_GDP1_-LEU1-T_PGK1_ | gggcgaattgggtaccatttatgtacccatgtataacc | |
|  |  |  |  | cgcctgttgctggtacagtttatcattatcaatactcgc | |
| pRS426-Li-cimA-mLeu2  Continued on next page | P_TEF1_-CYB2m-LI_*CimA*-T_ADH1_-P_GDP1_-CYB2m-*LEU2*- T_PGK1_ | *Kpn*I-digested pRS426- CYB2m-LI_CimA | - | - | |
|  |  | P_GDP1_-CYB2m-*LEU2*-T_PGK1_ | Plasmid pRS414- P_GDP1_-CYB2m-LEU2-T_PGK1_ | gggcgaattgggtaccatttatgtacccatgtataacc | |
|  |  |  |  | cgcctgttgctggtacagtttatcattatcaatactcgc | |
| **Plasmid** | **Description** | **Fragments for plasmid construction by assembling** | **PCR Template** | **Primer sequence** | |
| pRS426-Li-cimA-Leu2 | P_TEF1_-CYB2m-LI_*CimA*-T_ADH1_-P_GDP1_-*LEU2*-T_PGK1_ | *Kpn*I-digested pRS426- CYB2m-LI_CimA | - | - | |
|  |  | P_GDP1_-*LEU2*-T_PGK1_ | Plasmid pRS414- P_GDP1_-LEU2-T_PGK1_ | gggcgaattgggtaccatttatgtacccatgtataacc | |
|  |  |  |  | cgcctgttgctggtacagtttatcattatcaatactcgc | |
| pRS426-Li-cimA-mLeu4s | P_TEF1_-CYB2m-LI_*CimA*- P_GDP1_-CAT2m-*LEU4s*-T_PGK1_ | *Kpn*I-digested pRS426- CYB2m-LI_CimA | - | - | |
|  |  | P_GDP1_-CAT2m-*LEU4s*-T_PGK1_ | Plasmid pRS414- P_GDP1_- CAT2m-*LEU4s* - T_PGK1_ | gggcgaattgggtaccatttatgtacccatgtataacc | |
|  |  |  |  | cgcctgttgctggtacagtttatcattatcaatactcgc | |
| pRS426-Li-cimA-Leu5 | P_TEF1_-CYB2m-LI_*CimA*-T_ADH1_-P_GDP1_-*LEU5*-T_PGK1_ | *Kpn*I-digested pRS426- CYB2m-LI_CimA | - | - | |
|  |  | P_GDP1_-*LEU5*-T_PGK1_ | Plasmid pRS414- P_GDP1_-LEU5-T_PGK1_ | gggcgaattgggtaccatttatgtacccatgtataacc | |
|  |  |  |  | cgcctgttgctggtacagtttatcattatcaatactcgc | |
| pRS426-Li-cimA-Leu9 | P_TEF1_-CYB2m-LI_*CimA*-T_ADH1_-P_GDP1_-*LEU9*-T_PGK1_ | *Kpn*I-digested pRS426- CYB2m-LI_CimA | - | - | |
|  |  | P_GDP1_-*LEU9*-T_PGK1_ | Plasmid pRS414- P_GDP1_-LEU9-T_PGK1_ | gggcgaattgggtaccatttatgtacccatgtataacc | |
|  |  |  |  | cgcctgttgctggtacagtttatcattatcaatactcgc | |
| pRS426-Li-cimA-NFS1 | P_TEF1_-CYB2m-LI_*CimA*-T_ADH1_-P_GDP1_-*NFS1*-T_PGK1_ | *Kpn*I-digested pRS426- CYB2m-LI_CimA | - | - | |
|  |  | P_GDP1_-*NFS1*-T_PGK1_ | Plasmid pRS414- P_GDP1_-NFS1-T_PGK1_ | gggcgaattgggtaccatttatgtacccatgtataacc | |
|  |  |  |  | cgcctgttgctggtacagtttatcattatcaatactcgc | |
| pRS426-Li-cimA-L1L2  Continued on next page | P_TEF1_-CYB2m-LI_*CimA*-T_ADH1_-P_GDP1_-*LEU2*-T_PGK1_-P _PGK1_-*LEU1*-T_GPD1_ | *Sac*I-digested pRS426-Li-cimA-Leu2 | - | - | |
|  |  | P_PGK1_-*LEU1*-T_GPD1_ | Plasmid pRS424-LEU1 | gtatagcatgaggtcgctcgagctccggaagtaccttcaaagaatgg | |
|  |  |  |  | ccctcactaaagggaacaaaagctggaatctgtgtatattactgc | |
| **Plasmid** | **Description** | **Fragments for plasmid construction by assembling** | **PCR Template** | **Primer sequence** | |
| pRS414- P_PYK1_-NFS1-T_ADH2_ | P_PYK1_-*NFS1*-T_ADH2_ | *Xho*I-digested pRS414-P_PYK1_-T_ADH2_ (plasmid pRS414-P_GDP1_-T_PGK1_ is from ref. 6) | - | - | |
|  |  | *NFS1* | Plasmid pRS414- P_GDP1_-NFS1-T_PGK1_ | caaataaaacatcatcacacatgttgaaatcaactgctac | |
|  |  |  |  | gcaagcttctcagcaaaccctcaatgacctgaccatttg | |
| pRS426- CYB2m-LI_CimA-L1L2N1 | P_TEF1_-CYB2m-LI_*CimA*-T_ADH1_-P_GDP1_-*LEU2*-T_PGK1_-P_PGK1_-*LEU1*-T_GPD1_-P_PYK1_-*NFS1*-T_ADH2_ | *Sac*I-digested pRS426-Li-cimA-Leu2 | - | - | |
|  |  | P_PGK1_-*LEU1*-T_GPD1_ | Plasmid pRS424-LEU1 | gtatagcatgaggtcgctcgagctccggaagtaccttcaaagaatgg | |
|  |  |  |  | ccaaaatagtagcattaagctggaatctgtgtatattactgc | |
|  |  | P_PYK1_-*NFS1*-T_ADH2_ | Plasmid pRS414- P_PYK1_-*NFS1*-T_ADH2_ | cacagattccagcttaatgctactattttggagattaatc | |
|  |  |  |  | cactaaagggaacaaagctactaataggataaattataggaatttataac | |
| pRS423-KivD | P_TEF1_-*KivD*-T_PGK1_ | *Sac*I-digested pRS423 | - | - | |
|  |  | P_TEF1_-*KivD*-T_PGK1_ | Plasmid pRS425-*kivD* ^2^ | gttctagagcggccgccaccgcggtgggcagcaacaggcgcgttgga | |
|  |  |  |  | gggaacaaaagctggagctcaggaagaatacactatactggatctaaagagtaca | |
| pRS423- ARO10* | P_TEF1_-*ARO10**-T_ADH1_ (*ARO10** encodes Aro10p^I355Y^ ^7^) | *Sac*I-digested pRS423 | - | - | |
|  |  | P_TEF1_-*ARO10** | Plasmid pRS425-*aro10m*^2^ | ctagagcggccgccaccgcggtgggcagcaacaggcgcgttggac | |
|  |  |  |  | gaccaaacctctggcgaagaagtccaaagctctattttttatttcttttaagtgccgct | |
|  |  | T_ADH1_ | Genomic DNA of *S. cerevisiae* HZ848 | cttaaaagaaataaaaaatagagctttggacttcttcgc | |
|  |  |  |  | gaccatgattacgccaagcgcgcaattaaccctcactaaagggaacaaaagctggagctgagcgacctcatgctatacc | |
| pRS414- P_PYK1_-ADH7-T_ADH2_  Continued on next page | P_PYK1_-*ADH7*-T_ADH2_ | *Xho*I-digested pRS414-P_PYK1_-T_ADH2_ (plasmid pRS414-P_GDP1_-T_PGK1_ is from ref. 6) | - | - | |
|  |  | *ADH7* | Genomic DNA of *S. cerevisiae* HZ848 | aaataaaacatcatcacacatgctttacccagaaaaatttc | |
|  |  |  |  | gcttctcagcaaacccctatttatggaatttcttatcataatcg | |
| **Plasmid** | **Description** | **Fragments for plasmid construction by assembling** | **PCR Template** | **Primer sequence** | |
| pRS423-ADH7 | P_PYK1_-*ADH7*-T_ADH2_ | *Sac*I-digested pRS423 | - | - | |
|  |  | P_PYK1_-*ADH7*-T_ADH2_ | Plasmid pRS414- P_PYK1_-*ADH7*-T_ADH2_ | ggggatccactagttctagagcggccgccaccgcggtggaatgctactattttggagattaatc | |
|  |  |  |  | Ctaaagggaacaaaagctggagctgcagctactaataggataaattataggaatttataa | |
| pRS423-ADH7-KivD | P_PYK1_-*ADH7*-T_ADH2_-P_TEF1_-*KivD*-T_PGK1_ | *Sac*I-digested pRS423 | - | - | |
|  |  | P_PYK1_-*ADH7*-T_ADH2_ | Plasmid pRS423-ADH7 | ggggatccactagttctagagcggccgccaccgcggtggaatgctactattttggagattaatc | |
|  |  |  |  | gattcgcggtcctcgaaaattaaaagtccaacgcgcctgttgctgcagctactaataggataaattataggaatttataa | |
|  |  | P_TEF1_-*KivD*-T_PGK1_ | Plasmid pRS423-KivD | ctgccatatttcaatttgttataaattcctataatttatcctattagtagctgcagcaacaggcgcgttg | |
|  |  |  |  | gattacgccaagcgcgcaattaaccctcactaaagggaacaaaagctggagctcaggaagaatacactatactggatctaaagagtac | |
| pRS423-ADH7- ARO10*  Continued on next page | P_PYK1_-*ADH7*-T_ADH2_-P_TEF1_-*ARO10**-T_ADH1_ | *Sac*I-digested pRS423 | - | - | |
|  |  | P_PYK1_-*ADH7*-T_ADH2_ | Plasmid pRS423-ADH7 | ggggatccactagttctagagcggccgccaccgcggtggaatgctactattttggagattaatc | |
|  |  |  |  | gattcgcggtcctcgaaaattaaaagtccaacgcgcctgttgctgcagctactaataggataaattataggaatttataa | |
|  |  | P_TEF1_-*ARO10**-T_ADH1_ | Plasmid pRS423- ARO10* | caattttaattttgctgcctgccatatttcaatttgttataaattcctataatttatcctattagtagctgcagcaacaggcgcgttg | |
|  |  |  |  | gaccatgattacgccaagcgcgcaattaaccctcactaaagggaacaaaagctggagctgagcgacctcatgctatacc | |
| **Plasmid** | **Description** | **Fragments for plasmid construction by assembling** | **PCR Template** | **Primer sequence** | |
| pRS426- HOM3*-ILV1 | P_TEF1_-*HOM3**-T_ADH1_-P_FBA1_-*ILV1*-T_PYK1_ | pRS426-P_TEF1_-*HOM3**-T_ADH1_ | Plasmid pRS426-*HOM3** | ttgttgttcaagccagcggtgccagttggagagctccagcttttgttc | |
|  |  |  |  | cttggaaggttatacatgggtacataaatggagcgacctcatgctatac | |
|  |  | P_FBA1_ | Genomic DNA of *S. cerevisiae* HZ848 | gtagagtagctgacattttgaatatgtattacttgg | |
|  |  |  |  | gaacaaaagctggagctctccaactggcaccgctgg | |
|  |  | *ILV1* | Genomic DNA of *S. cerevisiae* HZ848 | gattctttttttaatatttcaagaatttttg | |
|  |  |  |  | caagtaatacatattcaaaatgtcagctactctactaaagc | |
|  |  | T_PYK1_ | Genomic DNA of *S. cerevisiae* HZ848 | ggtatagcatgaggtcgctccatttatgtacccatgtataac | |
|  |  |  |  | caaaaattcttgaaatattaaaaaaagaatcatgattgaatg | |
| pRS426- CYB2m-MJ_CimA | P_TEF1_-CYB2m-MJ_*CimA*-T_ADH1_ | pRS426-P_TEF1_-CYB2m-LI_*CimA*-T_ADH1_ | Plasmid pRS426- MJ_CimA | tcctcagagcgtctaagactagattgaacacaatccgcgcgtacggttctaccgttccaaaatccatggtaaggatatttgataca | |
|  |  |  |  | cttagacgctctgaggatagcagcctcacagttcttcgagatttttagtaaaggtttgtattttagcatttttgtaattaaaacttag | |
| pRS426- CYB2m-LI_CimA | P_TEF1_-CYB2m-LI_*CimA*-T_ADH1_ | pRS426-P_TEF1_-CYB2m-LI_*CimA*-T_ADH1_ | Plasmid pRS426- LI_CimA | tcctcagagcgtctaagactagattgaacacaatccgcgcgtacggttctaccgttccaaaatccacaaaagtagaaactcgattg | |
|  |  |  |  | cttagacgctctgaggatagcagcctcacagttcttcgagatttttagtaaaggtttgtattttagcatttttgtaattaaaacttag | |
| pRS426- CYB2m-GS_CimA | P_TEF1_-CYB2m-GS_*CimA*-T_ADH1_ | pRS426-P_TEF1_-CYB2m-LI_*CimA*-T_ADH1_ | Plasmid pRS426- GS_CimA | tcctcagagcgtctaagactagattgaacacaatccgcgcgtacggttctaccgttccaaaatccagccttgtaaaactctacgatac | |
|  |  |  |  | cttagacgctctgaggatagcagcctcacagttcttcgagatttttagtaaaggtttgtattttagcatttttgtaattaaaacttag | |

**Supplementary Table S2.** List of strains used in this study and their genotypes.

Continued on next page

| **Strain** | **Genotype** | **Plasmids** | **Source** |
| --- | --- | --- | --- |
| ***E. coli* strains** | | |  |
| *E. coli* DH5α | *supE*44 *lacU*169 (ϕ80*lacZ* ΔM15) *hsdR*17 *recA*1 *endA*1 *gyrA*96 *thi*-1 *relA*1 |  |  |
| ***S. cerevisiae* strains** | | |  |
| HZ848 | *MATα ura3*Δ *ade2-1 his3-11,15 leu2-3,112 can1-100 trp1-1* |  | 8 |
| *adh1*Δ | HZ848  *adh1*Δ | pRS426  pRS424 | This study |
| Reference  *adh1*Δ | HZ848  *adh1*Δ | pRS426  pRS424  pRS423 | This study |
| SS01 | HZ848 | pSS01 | This study |
| SS02 | HZ848 | pSS02 | This study |
| SS03 | HZ848 | pSS03 | This study |
| SS04 | HZ848 | pSS04 | This study |
| THR5 | HZ848  *adh1*Δ | pRS426-THR  pRS424 | This study |
| THR5* | HZ848  *adh1*Δ | pRS426-THR*  pRS424 | This study |
| HOM3m | HZ848  *adh1*Δ | pRS426-*HOM3**  pRS424 | This study |
| THRc | HZ848  *adh1*Δ | pRS424-LEU  pRS426-HOM3*-ILV1 | This study |
| THRm | HZ848  *adh1*Δ | pRS424-mLEU  pRS426-HOM3*-ILV1 | This study |
| MJ | HZ848  *adh1*Δ | pRS426-CYB2m-MJ_CimA  pRS424-mLEU | This study |
| LI | HZ848  *adh1*Δ | pRS426-CYB2m-LI_CimA  pRS424-mLEU | This study |
| GS | HZ848  *adh1*Δ | pRS426-CYB2m-GS_CimA  pRS424-mLEU | This study |
| LI-H3 | HZ848  *adh1*Δ | pRS426-Li-cimA-HOM3*  pRS424-mLEU | This study |
| LI-I1 | HZ848  *adh1*Δ | pRS426-Li-cimA-ILV1  pRS424-mLEU | This study |
| LI-cim | HZ848  *adh1*Δ | pRS426-Li-cimA_2copy  pRS424-mLEU | This study |
| LI-L1m | HZ848  *adh1*Δ | pRS426-Li-cimA-mLeu1  pRS424-mLEU | This study |
| LI-L1c | HZ848  *adh1*Δ | pRS426-Li-cimA-Leu1  pRS424-mLEU | This study |
| LI-L2m | HZ848  *adh1*Δ | pRS426-Li-cimA-mLeu2  pRS424-mLEU | This study |
| **Strain** | **Genotype** | **Plasmids** | **Source** |
| LI-L2c | HZ848  *adh1*Δ | pRS426-Li-cimA-Leu2  pRS424-mLEU | This study |
| LI-L4m | HZ848  *adh1*Δ | pRS426-Li-cimA-mLeu4s  pRS424-mLEU | This study |
| LI-L5 | HZ848  *adh1*Δ | pRS426-Li-cimA-Leu5  pRS424-mLEU | This study |
| LI-L9 | HZ848  *adh1*Δ | pRS426-Li-cimA-Leu9  pRS424-mLEU | This study |
| LI-N1 | HZ848  *adh1*Δ | pRS426-Li-cimA-NFS1  pRS424-mLEU | This study |
| LI-LL | HZ848  *adh1*Δ | pRS426-Li-cimA-L1L2  pRS424-mLEU | This study |
| LI-LLN | HZ848  *adh1*Δ | pRS426-CYB2m-LI_CimA-L1L2N1  pRS424-mLEU | This study |
| Kivd | HZ848  *adh1*Δ | pRS423-KivD  pRS424  pRS426 | This study |
| Aro10m | HZ848  *adh1*Δ | pRS423-ARO10*  pRS424  pRS426 | This study |
| ADH7 | HZ848  *adh1*Δ | pRS423-ADH7  pRS424  pRS426 | This study |
| A7KD | HZ848  *adh1*Δ | pRS423-ADH7-KivD  pRS424  pRS426 | This study |
| A7A10m | HZ848  *adh1*Δ | pRS423-ADH7-ARO10*  pRS424  pRS426 | This study |
| COM | HZ848  *adh1*Δ | pRS423-ADH7-ARO10*  pRS426-CYB2m-LI_CimA-L1L2N1  pRS424-mLEU | This study |

**Supplementary Figure S1.** Confirmation of the localization of GFP fused with or without the mitochondrial signal sequences. Sections were observed and photographed using a Zeiss LSM 5 Duo laser scanning microscope (Carl Zeiss; Jena, Germany). Overlaying of the bright-field image with that of GFP clearly indicates the localization.


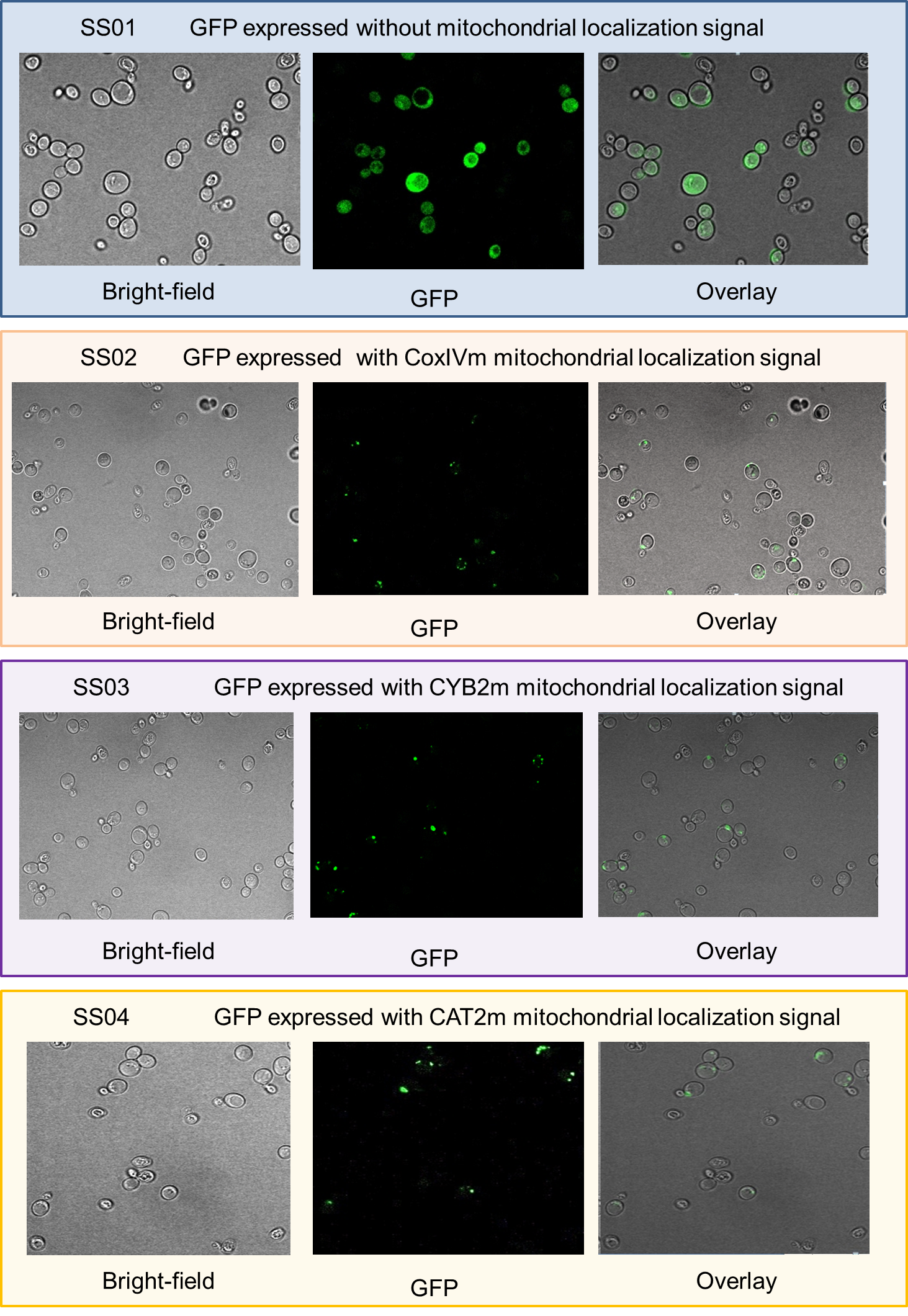


**Supplementary Figure S2.** Production of n-butanol and growth curve of strain COM. Micro-anaerobic batch fermentation of resting cells was performed in YPAD medium with 20 g/L glucose in 1.0 L Dasgip stirrer-pro® bioreactors (DasGip, Jülich, Germany) as described in the Materials and Method section. The initial OD of the cultures was 1.2 after inoculation from pre-cultures.

**
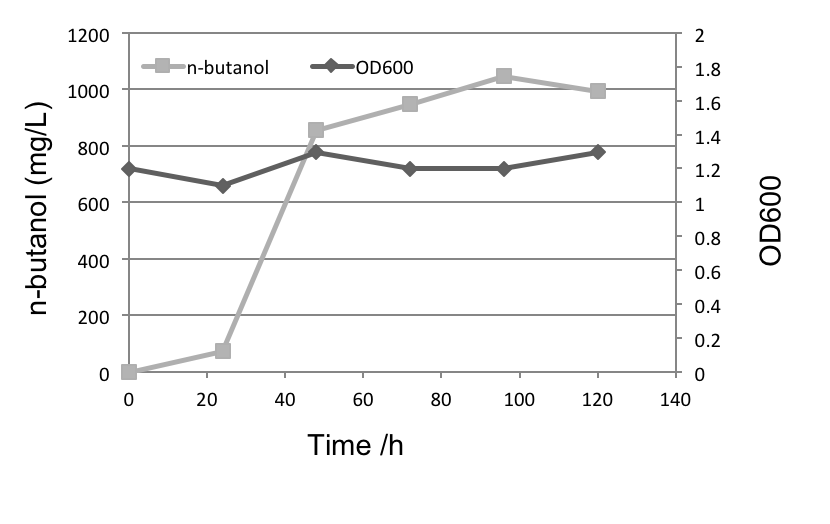
**

**References**

1. Lian, J., Si, T., Nair, N.U. & Zhao, H. Design and construction of acetyl-CoA overproducing *Saccharomyces cerevisiae* strains. *Metab. Eng.* **24**, 139-149 (2014).

2. Si, T., Luo, Y., Xiao, H. & Zhao, H. Utilizing an endogenous pathway for 1-butanol production in *Saccharomyces cerevisiae*. *Metab. Eng.* **22**, 60-68 (2014).

3. Sun, J. et al. Cloning and characterization of a panel of constitutive promoters for applications in pathway engineering in *Saccharomyces cerevisiae*. *Biotechnol. Bioeng.* **109**, 2082-2092 (2012).

4. Martin-Rendon, E., Farfán, M., Ramos, C. & Calderon, I. Isolation of a mutant allele that deregulates the threonine biosynthesis in *Saccharomyces cerevisiae*. *Curr Genet* **24**, 465-471 (1993).

5. Beltzer, J.P., Morris, S.R. & Kohlhaw, G.B. Yeast *LEU4* encodes mitochondrial and nonmitochondrial forms of alpha-isopropylmalate synthase. *J. Biol. Chem.* **263**, 368-374 (1988).

6. Du, J. Metabolic engineering of *Saccharomyces cerevisiae* for efficient ethanol production from pentose sugars. Ph.D. Thesis, University of Illinois at Urbana-Champaign, IL (2011).

7. Kneen, M.M. et al. Characterization of a thiamin diphosphate-dependent phenylpyruvate decarboxylase from *Saccharomyces cerevisiae*. *FEBS J.* **278**, 1842-1853 (2011).

8. Shao, Z., Zhao, H. & Zhao, H. DNA assembler, an *in vivo* genetic method for rapid construction of biochemical pathways. *Nucleic Acids Res.* **37**, e16 (2009).
